# Warfarin-induced toxic epidermal necrolysis in combination therapy of Henoch-Schönlein purpura nephritis: a case report

**DOI:** 10.1186/s12882-017-0648-9

**Published:** 2017-07-14

**Authors:** Katsuaki Kasahara, Yoshimitsu Gotoh, Yoshiyuki Kuroyanagi, China Nagano

**Affiliations:** Department of Pediatric Nephrology, Japan Red Cross Nagoya Daini Hospital, 2-9, Myoumi-chou, Shouwaku, Nagoya-shi, Aichi-ken 466-8650 Japan

**Keywords:** Toxic epidermal necrolysis, Warfarin potassium, Lymphocyte transformation test, Henoch–Schönlein purpura nephritis, Case-report

## Abstract

**Background:**

Toxic epidermal necrolysis (TEN) is a rare life-threatening condition almost exclusively attributed to drugs. The main etiologic factors for TEN are sulphonamides, anticonvulsants, and antibiotics; however, there are no published reports of warfarin causing TEN.

**Case presentation:**

We present the case of a 3-year-old patient who developed TEN while receiving treatment for Henoch–Schönlein purpura nephritis (HSPN). With multiple-drug therapy comprising prednisolone, mizoribine, dipyridamole, and warfarin, it is difficult to detect which drug is the causative agent. While in most cases, diagnosis of the causative drug is based on clinical history without a lymphocyte transformation test (LTT), we performed the test three times and identified the causative drug as warfarin at the late phase. We continued HSPN treatment without warfarin, and results showed good renal function without life-threatening complications.

**Conclusion:**

To our knowledge, this is the first report about TEN caused by warfarin. Repeated LTTs could be useful for identifying TEN-causative drugs even in the late phase.

## Background

Toxic epidermal necrolysis (TEN) is a rare life-threatening condition almost exclusively attributed to drugs. More than 200 drugs have been associated with TEN, the most common ones being sulphonamides, anticonvulsants, and antibiotics [1, 2], but there are no published reports of warfarin causing TEN. Pinpointing the exact causative agent may be difficult when multiple medications are used concomitantly. Lymphocyte transformation test (LTT) measures the proliferation of T cells to a drug in vitro from which one concludes to a previous in vivo reaction due to a sensitization. This idea of the LTT has been confirmed by the generation of drug-specific T-cell clones and the finding that drugs can directly interact with the T-cell receptor. LTT is a safe and reproducible method for diagnosing drug hypersensitivity; however, in TEN, it must be performed within the early phase (the first week after onset of rash) to aid diagnosis [3]. We observed warfarin-induced TEN during combination therapy of Henoch–Schönlein purpura nephritis (HSPN), and LTT results were positive at the late phase.

## Case presentation

**Fig. 1:**
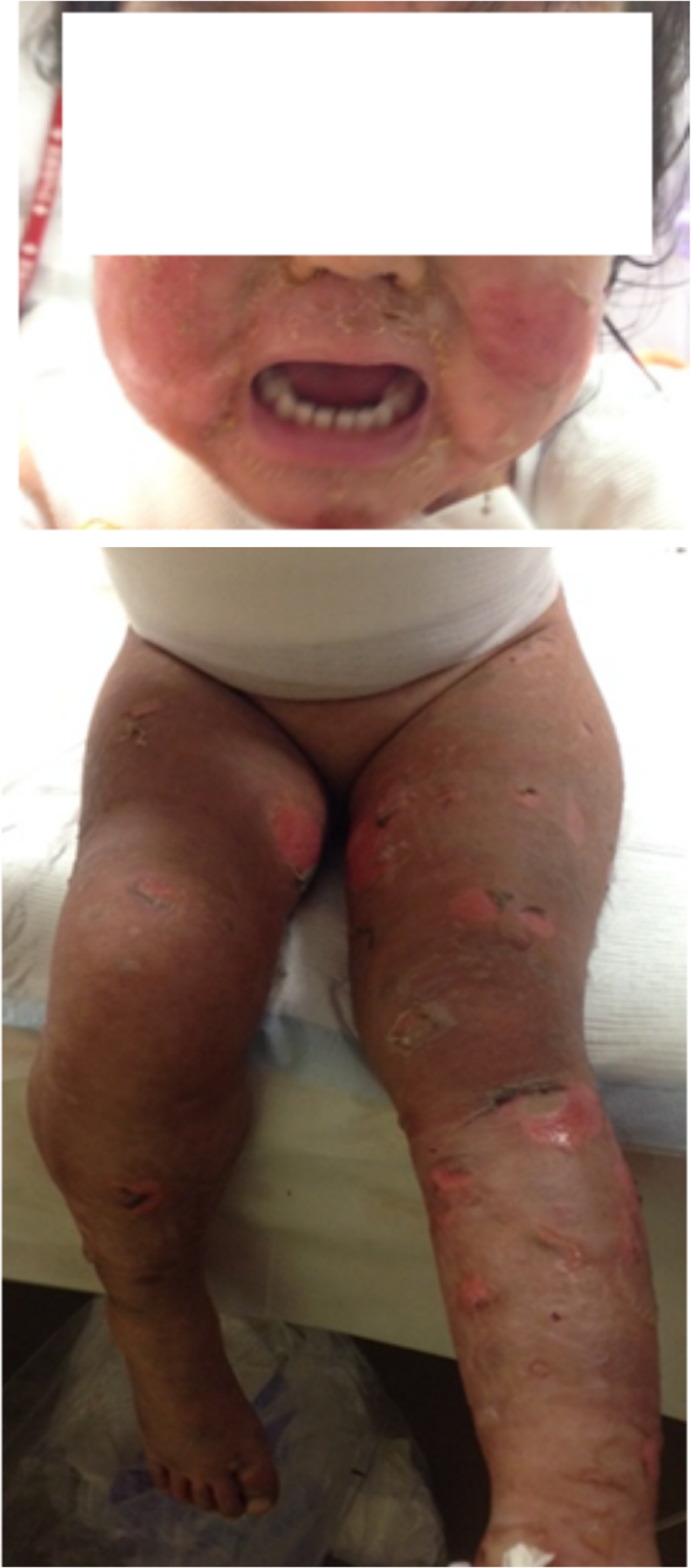
Face and legs with macular and exfoliative skin lesions

A three-year-old girl was diagnosed with HSPN classified grade IV by the International Study of Kidney Diseases in Children (ISKDC). The patient received plasmapheresis (PP) and methylprednisolone pulse therapy, followed by combination therapy comprising prednisolone, mizoribine, dipyridamole, and warfarin. On the 13th day of combination therapy, we discontinued prednisolone and mizoribine because the patient had developed erythema, abrasions, and blisters on her palms and legs. Three days later, she developed a high fever and her skin condition deteriorated. At presentation, she had a fever (40 °C), mucous membrane involvement with mild ocular lesions, and Nikolsky sign over 80% of her body surface area (BSA). At that time, we diagnosed TEN clinically, and her Score of Toxic Epidermal Necrolysis (SCORTEN) was 2 (Fig. 1). We discontinued dipyridamole and warfarin.

Blood tests revealed the following: white blood cell (WBC) count, 8600 cells per μL; platelet count, 36.5 × 10^4^ cells per μL; creatinine (Cr), 0.30 mg/dL; C-reactive protein (CRP), 1.36 mg/dL (normal, < 0.5 mg/dL); AST, 23 IU/L (normal, 10–40 IU/L); ALT, 13 IU/L (normal, 5–40 IU/L); d-dimer, 20.3 μg/mL (normal, < 1.0 μg/dL); mycoplasma pneumonia IgM, < 40 times (normal). Urinary test results were as follows: urine sediment, 1–4 μL (normal, < 5 μL) for WBCs, and 30–49 μL (normal, < 5 μL) for red blood cells. Urine biochemistry results showed a urinary protein/creatinine ratio of 0.5 g/gCre (normal, < 0.2).

**Fig. 2:**
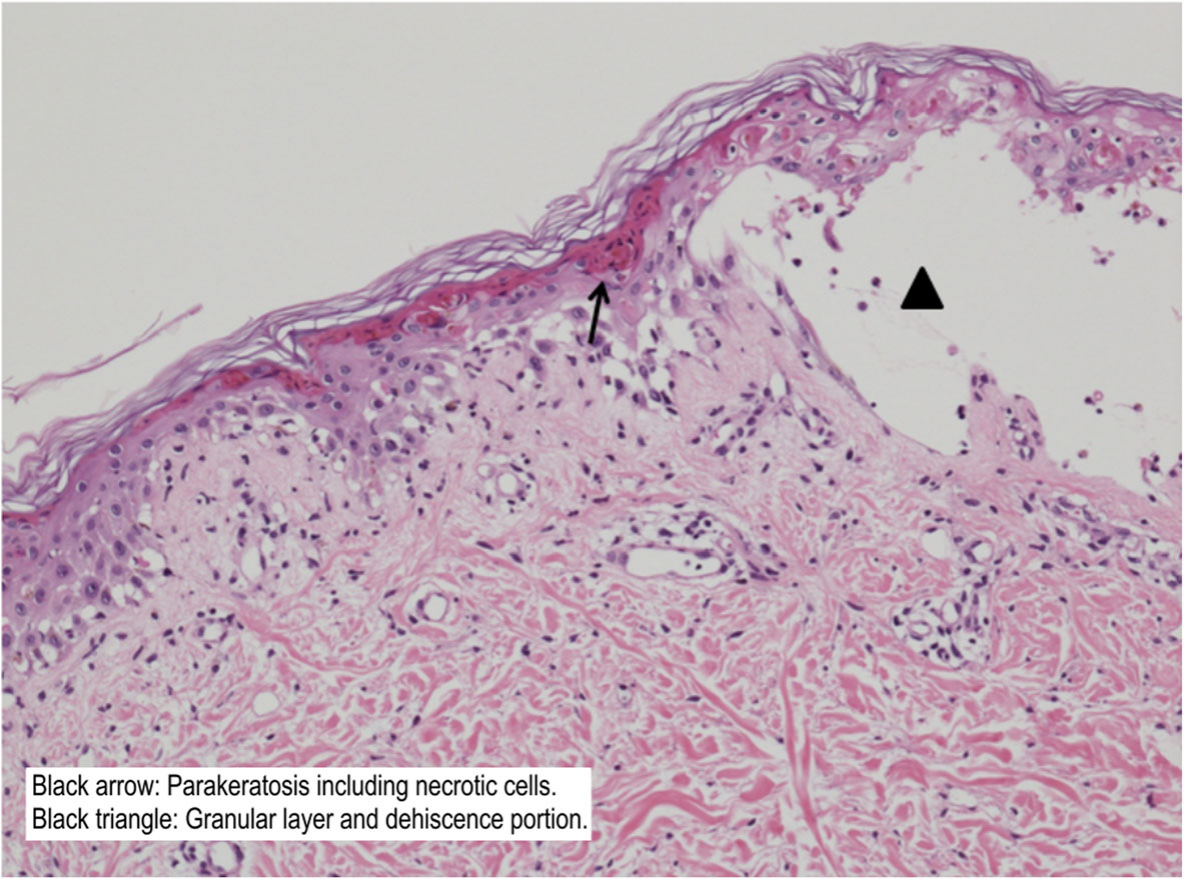
Histopathological examination of a skin biopsy taken from the outside of the right thigh. The examination revealed necrotic keratinocytes and papillary dermal edema

We performed a skin biopsy (Fig. 2) on the first and second days of hospitalization. Skin biopsy specimens showed interface dermatitis with apoptotic bodies in the epidermis and a superficial perivascular infiltrate of mononuclear cells with some polymorphonuclear leukocytes.

**Fig. 3:**
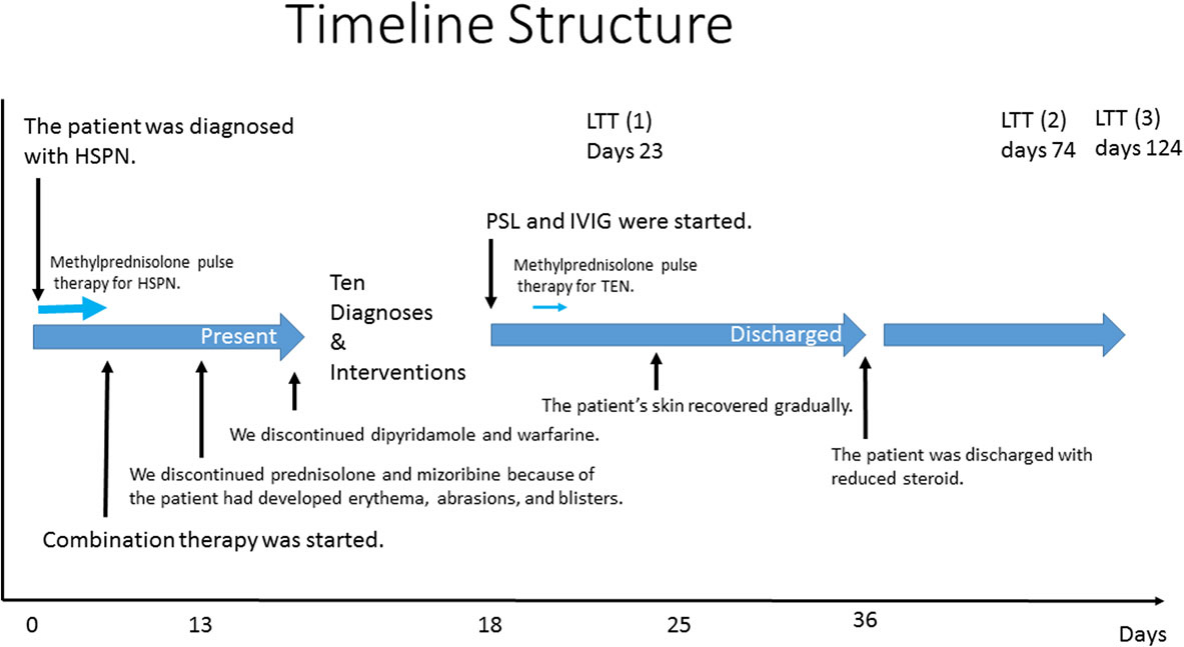
Timeline of case. HSPN, Henoch–Schönlein purpura nephritis; TEN, Toxic Epidermal Necrolysis; LTT, lymphocyte transformation test; Combination therapy comprising prednisolone, mizoribine, dipyridamole, and warfarin; IVIG, intravenous immunoglobulins

We restarted prednisone treatment (1 mg/kg/day), and added intravenous immunoglobulins (IVIG; 400 mg/kg/day) for 5 days from the second day of hospitalization. On the 5th day of hospitalization, because the patient had no infectious disease but her condition was not improving, we added methylprednisolone pulse therapy (30 mg/kg/day) for 3 days, followed by prednisone. The patient’s skin recovered gradually, and Nikolsky sign decreased to less than 10% of BSA. On the 18th day, the skin was almost normal, and the patient was discharged with steroid therapy (1 mg/kg/day) (Fig. 3). One month later, the dose of prednisone was reduced to 1 mg/kg every other day, and there were no life-threatening complications with use of PSL. LTT for drugs (mizoribine, dipyridamole, and warfarin) was performed three times: once during the early phase (day 23) for mizoribine, and twice during the late phase (days 74 and 124) for all three drugs. The LTT results were as follows: Day 23 (control 512 cpm) for mizoribine, stimulation index (SI) was negative (0.8, normal, < 1.8); day 74 for mizoribine (control 221 cpm), SI was negative (0.4, normal, < 1.8); day 74 for dipyridamole, SI was negative (0.3, normal, < 1.8); day 74 for warfarin, SI was negative (0.7, normal, < 1.8); day 124 for mizoribine (control 230 cpm), SI was negative (0.5, normal, < 1.8); day 124 for dipyridamole, SI was negative (0.5, normal, < 1.8); day 124 for warfarin, SI was positive (5.3, normal, < 1.8).

Only the late phase day 124 LTT result for warfarin SI was positive, while day 74 of SI was negative. At the late phases, we stopped using prednisone 2 days before the day 74 LTT and 5 days before the day 124 LTT.

## Discussion

We encountered warfarin-induced TEN in HSPN combination therapy; to our knowledge, this is the first report of this phenomenon. The LTT result was only positive for warfarin at the late/recovery phase in TEN when steroids had been stopped prior to the test.

We could not find reports in English of warfarin-induced TEN or Stevens-Johnson syndrome (SJS) using MEDLINE. The literature of TEN case series is summarized in Table 1. In three of the five reports, skin detachment occurs in over 30% of BSA. Most patients are clinically diagnosed as TEN caused by drugs without LTT (Table 1). Corticosteroids and/or IVIG are used for many TEN patients (Table 1). The main supportive care is the same as that for major burns and includes wound care, fluid and electrolyte management, and nutritional support (Table 1). A literature review revealed that drugs suspected to be responsible for TEN included sulphonamides, anticonvulsants, and antibiotics (Table 1). In contrast with TEN in adults, allopurinol and nevirapine were not identified as causative agents in children. Drug prescription patterns may be responsible for the changes in drugs suspected of causing TEN.

**Table 1:** Literature of TEN causes (case series of five or more pediatric patients 2009–2015)

Author (year)	Number of pediatric patients	Age (years)	SCORTEN (mean ± SD)	BSA affected (%; mean ± SD)	Rash days from drug intake (days)	Etiology (%)	LTT	Treatment (%)	Outcome
Quirke et al. (2015) [7]	*N* = 41 (BSA > 30%: *n* = 23, BSA ≤ 30%; *n* = 16, unknown; *n* = 2:)	11.2 ± 4.6	1.4 ± 0.7	39.7 ± 26.0	ND	Drugs (90%: anticonvulsants, antibiotics, NSAIDS, others), mycoplasma pneumoniae (5%), unknown (5%)	ND	Corticosteroids (54%), IVIG (17%), supplemental nutritional support (90%), required mechanical ventilation (51%)	Mortality (0%)
Hamilton et al. (2013) [8]	*N* = 10 (BSA 20–30%: *n* = 4, BSA > 30%: *n* = 4, unknown: *n* = 2)	6.6 ± 3.9	1.7 ± 0.5	42.2 ± 20.6	ND	Drugs (70%: anticonvulsants, antibiotics, others), mycoplasma infection (20%), unknown (10%)	ND	IVIG (80%), corticosteroids (20%), antibiotics (60%)	Mortality (0%)
Finkelstein et al. (2011) [9]	*N* = 55 (TEN: *n* = 5, SJS: *n* = 47, SJS/TEN: *n* = 3)	9.6 ± 4.8	ND	ND	ND	Drugs (anticonvulsants: 29%, antibiotics: 20%, chemotherapy drugs: 2%), infections (mycoplasma pneumonia: 22%, herpes simplex virus: 9%), undetermined (18%)	ND^a^	Supportive care (100%), antibiotics (67%), antiviral (38%), corticosteroids (40%), IVIG (38%), corticosteroids plus IVIG (14%)	Mortality (2%)
Ferradiz et al. (2011) [10]	*N* = 14 (TEN: *n* = 6, SJS: *n* = 8)	10.4 (range: 1–17)	ND	60 (range: 10–96)	16.5 (1–30)	Suspicious drugs (anticonvulsants: 50%, antiviotics: 36%, NSAIDS: 21%)	ND	Supportive care (100%), corticosteroids: 1-2 mg/kg/day, 9 days (86%), IVIG: 1-2 g/kg/day, 3 days (29%), silversulfadiazine dressings (71%)	Mortality (7%)
Levi et al. (2009) [1]	*N* = 80 (TEN: *n* = 27, SJS: *n* = 21, SJS/TEN: *n* = 32)	6.2 (IQR: 3.7–9.9)	ND	20 (range: 10–40)	6 (range; 1–17)^b^	Drugs (anticonvulsants: 30%, antiviotics: 40%, others), mycoplasma pneumoniae (9%)	ND	ND	Mortality (7.5%)

We excluded reports describing <10 patients, and those that did not provide data about causative drugs or treatments

All reports published between 2009 and 2015 in English describing TEN in children were retrieved using MEDLINE

*Abbreviations*: *BSA* body surface area, *SCORTEN* Score of Toxic Epidermal necrolysis, *ND* no data, *TEN* Toxic Epidermal Necrolysis, *SJS* Stevens-Johnson Syndrome, *LTT* lymphocyte transformation test, *IQR* interquartile range, *NSAID* nonsteroidal anti-inflammatory drugs

^a^15 children underwent skin biopsies

^b^Days between acetaminophen initiation and onset of disease

Diagnosis for TEN and its causative drugs is usually based on clinical history without allergological tests such as LTT. We performed a skin biopsy as well as LTT, which is a safe and reproducible test to assess activation of drug-specific T cells in vitro; however, in cases of TEN/SJS, it must be performed within the early phase (first week after onset of the rash) to aid diagnosis [3]. Our LTT result was positive during the late phase, similarly to drug-induced hypersensitivity syndrome/drug rash and eosinophilia with systemic symptoms (DIHS/DRESS). In DIHS/DRESS patients, the regulatory T cells dramatically increase in frequency in the acute stage, and return to normal upon recovery; this change was specifically observed in patients with DIHS but not in TEN drug reactions, indicating that an increase in regulatory T cells may contribute to negative LTT results at the acute stage but positive results at the late phase [3]. While we did not examine T cells, our findings suggest that not only cross-reactivity of T cells but also the strength of the original reaction observed at the acute stage would determine the persistence of positive LTT results [3]. Our LTT result for TEN may have been positive even at the early phase because of the strength of the TEN severities (SCORTEN 2). We ruled out DIHS/DRESS because the patient’s symptoms were not accompanied by peripheral eosinophilia ≥1.5 × 10^9^ cells/L, atypical lymphocytes, or systemic abnormalities. We also ruled out SJS, because her affected skin area was over 30% of BSA.

Systemic prednisolone has been long thought to have an inhibitory effect on the SI value. Pichler and Tilch [4] recommended that the LTT be performed with blood from patients receiving <0.2 mg/kg prednisolone. At late phase, our patient was receiving prednisolone (1 mg/kg every other day). Duration periods without steroids (two and 5 days prior to each late-phase LTT) may affect the LTT.

Since the LTT results were negative for mizoribine and dipyridamole, we re-prescribed them with no side effects to the patient. Stopping warfarin therapy at the beginning of HSPN therapy should not have affected urinary test results and kidney function, because warfarin should only be used for 2 months by protocol. Because we could use mizoribine, dipyridamole, and prednisolone again for HSPN therapy without warfarin, the patient’s urine values returned to normal without kidney injury.

Finally, we could rapidly withdraw the TEN-causative drug and add steroids, IVIG, and methylprednisolone pulse therapy with supportive care, which have been shown to contribute to a favorable outcome [3, 5]. The use of steroids and IVIG has not been evaluated in clinical trials, and remains controversial. Schneck et al. [5] found that a short course of prednisone (1–2 mg/kg/day) may not be harmful, and may even have a beneficial effect if administered early in the course of the disease. In their study, the mortality rate was 34% in the group treated with IVIG alone, but 18% in the group treated with IVIG and steroids. In another study, Lee et al. [6] found that the odds ratio of death for patients treated with steroids compared with patients treated with supportive care alone was 0.6 (95% confidence interval: 0.3–1.0), suggesting a potential benefit for treatment with steroids.

Published cases of TEN are shown in Table 1. In over 70% of cases, the etiologic cause was drugs; most therapies consisted of corticosteroids and/or IVIG. In our case, the appearance of TEN symptoms dated from the causative drug intake, is relatively late compared with symptom presentation in published literature (Table 1). It is possible that the exposure to steroids before the onset of TEN prolonged the latency and progression of the disease [6].

## Conclusion

To our knowledge, this is the first report of warfarin-induced TEN. While in most cases, identification of the causative drug is based on the clinical history without LTT (Table 1), we performed the test three times and detected the causative drug during the late phase. When HSPN therapy was started without warfarin, proteinuria disappeared and renal function was not compromised. Repeated LTTs could be useful for detecting the causative drug even in the late phase.

## Abbreviations

BSA: Body surface area
Cr: Creatinine
CRP: C-reactive protein
DIHS: Drug-induced hypersensitivity syndrome
DRESS: Drug rash and eosinophilia with systemic symptoms
HSPN: Henoch–Schönlein purpura nephritis
ISKDC: International Study of Kidney Diseases in Children
IVIG: Intravenous immunoglobulins
LTT: Lymphocyte transformation test
PP: Plasmapheresis
PT-INR: Prothrombin time-international normalized ratio
SCORTEN: Score of Toxic Epidermal Necrolysis
SI: Stimulation index
SJS: Stevens-Johnson Syndrome
TEN: Toxic epidermal necrolysis
WBC: White blood cell
